# *Crossword*: A data-driven simulation language for the design of genetic-mapping experiments and breeding strategies

**DOI:** 10.1038/s41598-018-38348-y

**Published:** 2019-03-13

**Authors:** Walid Korani, Justin N. Vaughn

**Affiliations:** 1 Center for Applied Genetic Technologies, The University of Georgia, Athens, GA 30602 USA; 2 United States Department of Agriculture, Athens, GA 30602 USA

## Abstract

Quantitative genetic simulations can save time and resources by optimizing the logistics of an experiment. Current tools are difficult to use by those unfamiliar with programming, and these tools rarely address the actual genetic structure of the population under study. Here, we introduce *crossword*, which utilizes the widely available re-sequencing and genomics data to create more realistic simulations and to reduce user burden. The software was written in R, to simplify installation and implementation. Because *crossword* is a domain-specific language, it allows complex and unique simulations to be performed, but the language is supported by a graphical interface that guides users through functions and options. We first show *crossword*’s utility in QTL-seq design, where its output accurately reflects empirical data. By introducing the concept of levels to reflect family relatedness, *crossword* can simulate a broad range of breeding programs and crops. Using levels, we further illustrate *crossword*’s capabilities by examining the effect of family size and number of selfing generations on phenotyping accuracy and genomic selection. Additionally, we explore the ramifications of large phenotypic difference between parents in a QTL mapping cross, a scenario that is common in crop genetics but often difficult to simulate.

## Introduction

The simulation of controlled crosses has been useful in comparing breeding strategies (reviewed in^1^) and experimental design of genetic mapping studies^2,3^. Current open-source packages have expanded the realism and utility of simulated scenarios by incorporating elements such as variation in recombination frequencies, novel transgenic approaches, and genomic selection^4,5^. Still, this realism comes at a significant cost, both, in terms of computational speed and difficulty of use. We have developed a simulation platform that uses real genotypic data and emerging genomics data in order to make realistic simulations much easier to implement. The platform, called “crossword”, is essentially a domain-specific language that, when executed, is interpreted into and executed by the R statistical programming environment. This layer of abstraction allows users to focus less on the mechanics of implementing the simulation and more on their actual experimental goals and/or breeding ideas. In addition, this precise syntax allows *crossword* to take full advantage of the familial structure unique to controlled crosses.

While the capabilities of simulators such as AlphaSim^4^ are substantial, it and other similar packages still do not take full advantage of empirical genomics data that could substantially simplify both the computation and usage of simulation for plant and animal breeders [https://popmodels.cancercontrol.cancer.gov/gsr/]. For example, the first step in most simulation frameworks usually involves generating a large-number of founder haplotypes via coalescent simulation with recombination. Parents in the pedigree are then randomly selected from these founders. In the very near future, breeding programs will also have access to genome sequences for many founder lines within a breeding program as well as extensive genotypic information to impute this information onto their progeny populations. Thus, initial coalescent simulations that create founder sequences are often irrelevant and even an obstacle for many breeders, who already have resequencing data for the parent lines in their study and would prefer to use that information. Unrealistic simplifying assumptions about founder structure can also have substantial implications on predicting breeding results^6^. Although, some of these simulators offer a means to supply parental genotypes, this functionality is very difficult to implement in practice. Only recently have some researchers begun to build in functionality for direct import of genotypic data^6^. Indeed, to our knowledge, no simulation framework fully supports the integration of empirical data, such as genome sequences and annotations, that are currently available for most crop species [https://popmodels.cancercontrol.cancer.gov/gsr/]. *Crossword* supports direct import of FASTA, GFF, VCF, and HAPMAP file formats and uses this information to substantially simplify the user experience and make simulations as case-specific as possible.

**Figure 1:**
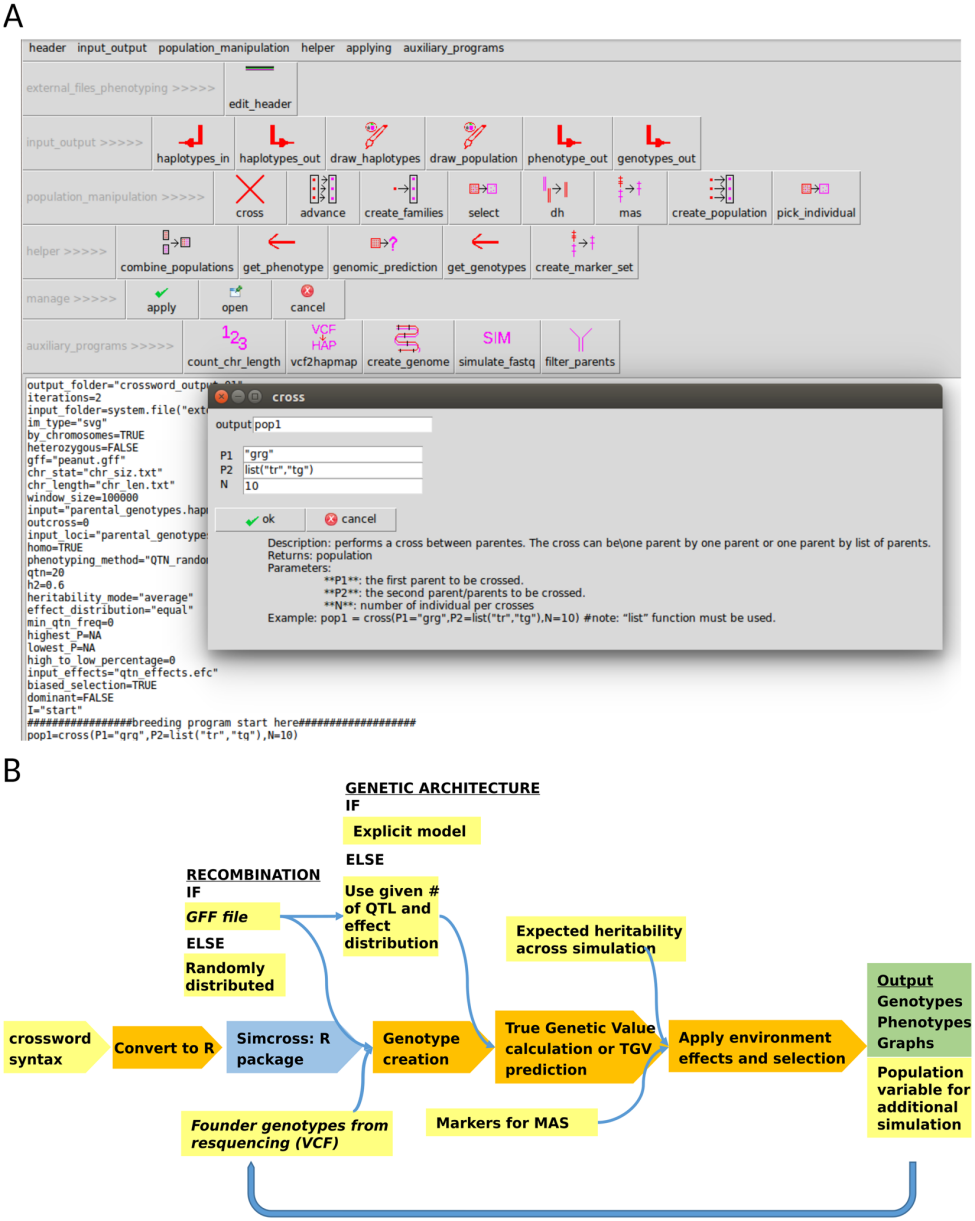
Syntax and execution of a crossword run. (**A**) User interface layout and editing window for crossword syntax. (**B**) Schematic of crossword simulation execution. Yellow color indicates arguments or files taken from user. Blue fill indicates an external dependency. Italic font indicates major source of external genomic data.

*Crossword* has three anticipated tiers of usage: (1) Most users will initially interact with *crossword* via a semi-graphical editor run under gWidgets/tcltk, a lightweight R windowing environment (Fig. 1A). This interface frees users of having to thoroughly familiarize themselves with the functions and parameters prior to use. Commands are automatically generated based on either the displayed defaults or the values supplied to the text-box entries. (2) All *crossword* runs generate a plain-text, run file. Therefore, those who wish to automate the exploration of a large-range of parameters can manipulate this run file by manually replacing parameters of interest with variable names and supplying an additional paremeter file. (3) All R functions underlying *crossword* are available and well-documented for those who have experience in R and need to directly modify or expand a behavior.

In what follows, we describe some of hurdles to realistic breeding and genetic mapping simulations. We report on the methods by which *crossword* simplifies these problems, and we give examples of how the resultant realism can be critical to major aspects of experimental design and interpretation.

## Results and Discussion

### Graphical interface and output facilitate “out-of-the-box” usability and utility

The *crossword* run script is interpreted into a set of R functions; thus, *crossword* can be installed on any system capable of running R. *Crossword* allows the user to submit a text file defining a small number of input files and describing the crossing scheme in a simple syntax (Fig. 1A and Fig. S2). A semi-graphical interface guides users to the appropriate functions and supplies “point-of-use” documentation.

The founder haplotypes in any *crossword* simulation are supplied by the user as either a VCF or HapMap file (Fig. 1B). Large population sizes coupled with numerous breeding cycles can be computationally burdensome if using genome-wide polymorphism data. Instead, as with most current simulators, *crossword* only tracks recombinations and the resultant configuration of founder haplotypes^7^.

Any variant in the supplied VCF file can be selected as a simulated quantitative trait nucleotide (QTN), which are causal variants with some genetic effect on the phenotype. For example, if the genetic architecture consists of 30 QTN, then these QTN are sampled from across population-wide polymorphisms, and, thus, a single cross may or may not be segregating for all 30 QTN. If a user wants all specified QTNs to be segregating in a biparental cross, they can filter VCF files to the appropriate parents. (*crossword* supplies auxiliary scripts to perform this and other capabilities, if needed). When a phenotyping or genotyping step is performed on a population, genetic coordinates are converted to genomic coordinates, the appropriate SNPs for a given founder haplotype are assigned, and phenotypes calculated. Given the computational demand on this step, *crossword* uses Rcpp/C+ + to optimize the speed of operations ~60-fold.

**Figure 2:**
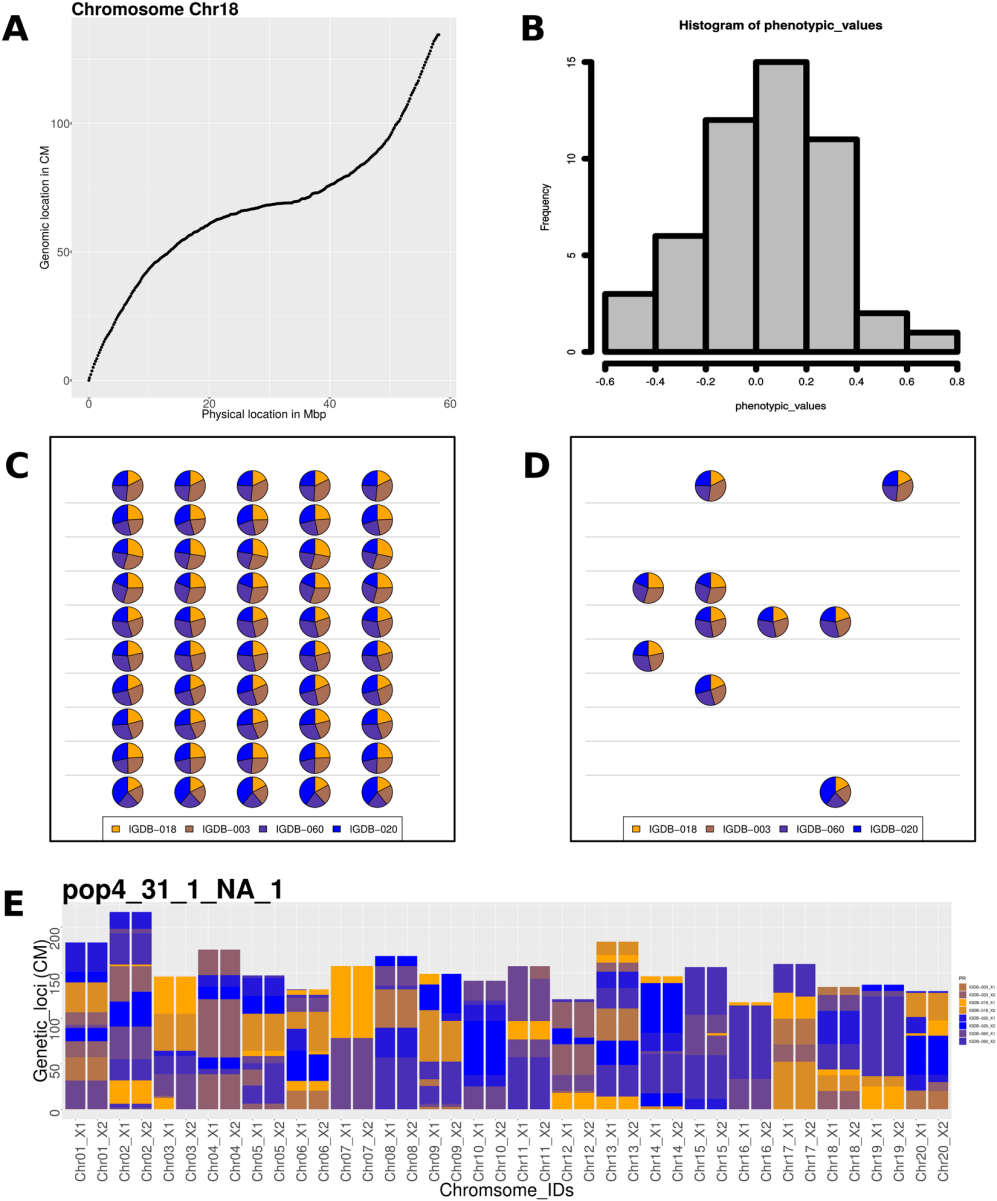
Graphical output examples. (**A**) Relationship of physical distance (Mb) to genetic distance (cM) based on gene density in a sliding widows of 10 kbp. (**B**) Phenotypic distribution of a particular population. (**C**) Parental contributions of the population resulting from 4 founders intercrossed and advanced for F5 generations. The population is composed 10 families, each having 5 individuals. (**D**) The selected highest ten individual based on phenotypic values. (**E**) Chromosomal representations of an individual from one of the above families. X1 and X2 represent the homologous chromosomes. Identical-by-descent regions are color coded based on founding donors.

Users are able to generate graphical output depicting haplotypes for any individual at any generation, genetic-by-physical distance functions for each chromosome, genetic breeding values relative to phenotypic simulation, and dense plots of population structure and founder contributions for a population (Fig. 2). Numerous output formats are supported including SVG and PNG.

### Gene-density approximation and crossover frequencies

One of *crossword*’s most useful characteristics relates to its usage of pre-existing genomic information to increase the realism of simulations without overburdening the user. As an example, other simulation suites, if they allow variation in recombination along the genome, require the user to manually create genetic-by-physical distance matrices in order to simulate this behavior. This requires access to data from a particular mapping study or substantial effort to align markers to the genome. Although *crossword* accepts such empirical data in order to model recombination functions, *crossword* can alternatively use gene annotation data. This later approach is based on the broadly observed correlation between gene density and recombination rate^8^. Gene density can be easily acquired from a GFF file available with almost all genome releases.

**Figure 3:**
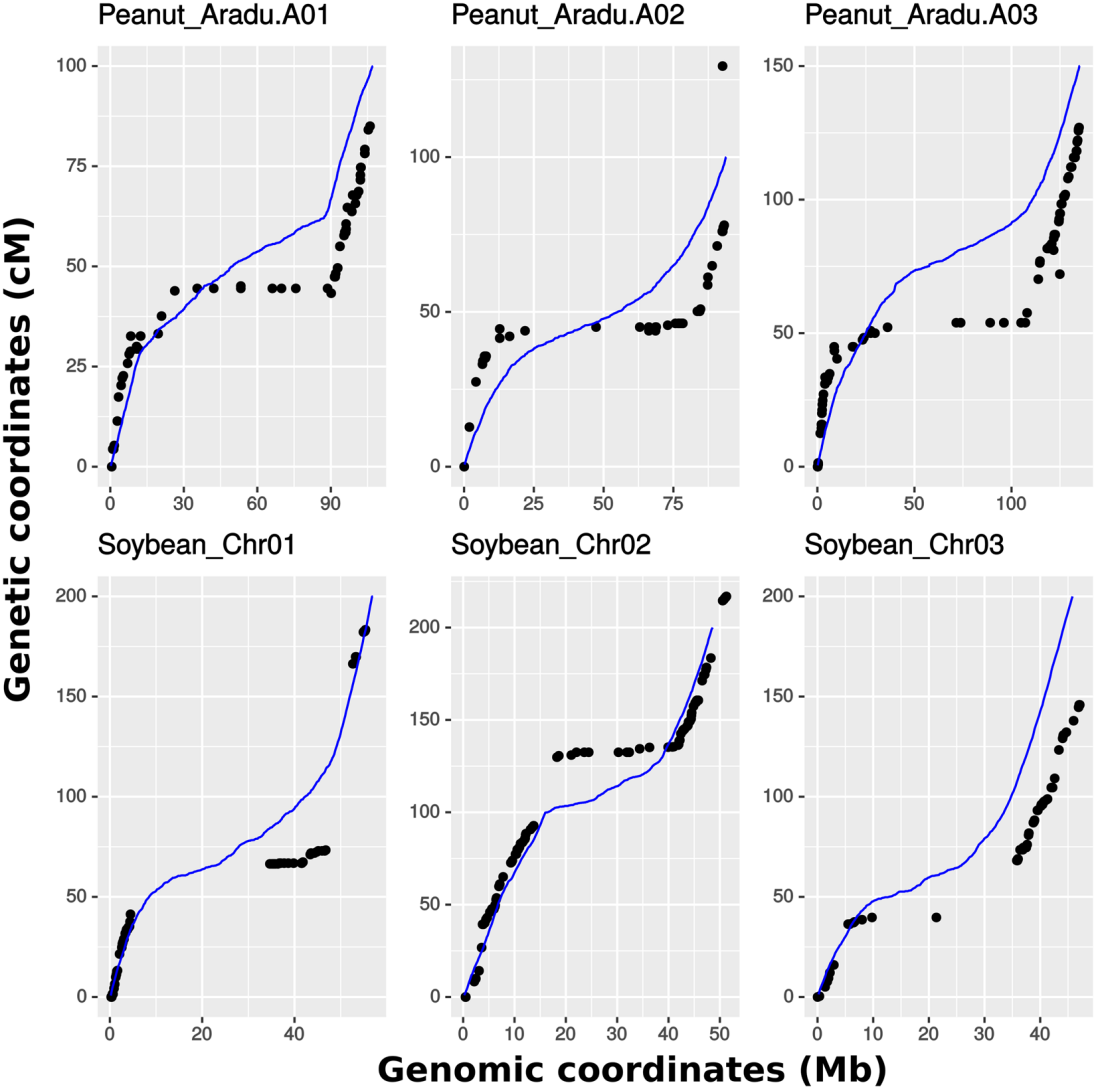
Recombination modeling in crossword. The relationship between genetic and physical distance is shown for three randomly selected chromosomes from two species. Points are based on empirical data from biparental populations whereas lines represent the function used by crossword based on gene density. Plots for all chromosomes are shown in Fig. S3.

We compared the performance with two genetic maps, one in peanut and one in soybean (David Bertioli and Ben Stewart-Brown, personal communication). Given that these represent only two populations within each species, the fit generally approximates the maps with the exception that the centromere displays slightly less recombination than predicated by gene density alone (Fig. 3). As seen in prior work^9^, other factors – methylation status, GC content, polymorphism density, structural variation, among others - contribute to recombination rate. Generally, data for these factors are less readily available and so we have not incorporated these in the current version.

### Empirical and *crossword* results for QTL-seq on oligogenic disease resistant trait

QTL-seq is an emerging genetic mapping strategy that combines bulk segregant analysis with next-generation sequencing^10^. In order to examine *crossword*’s capacity to reflect actual experiment results,we simulated one such previously published QTL-seq experiment and included all parameters relevant to that population and its development^11^ (and Joshua Clevenger, personal communication).

**Figure 4:**
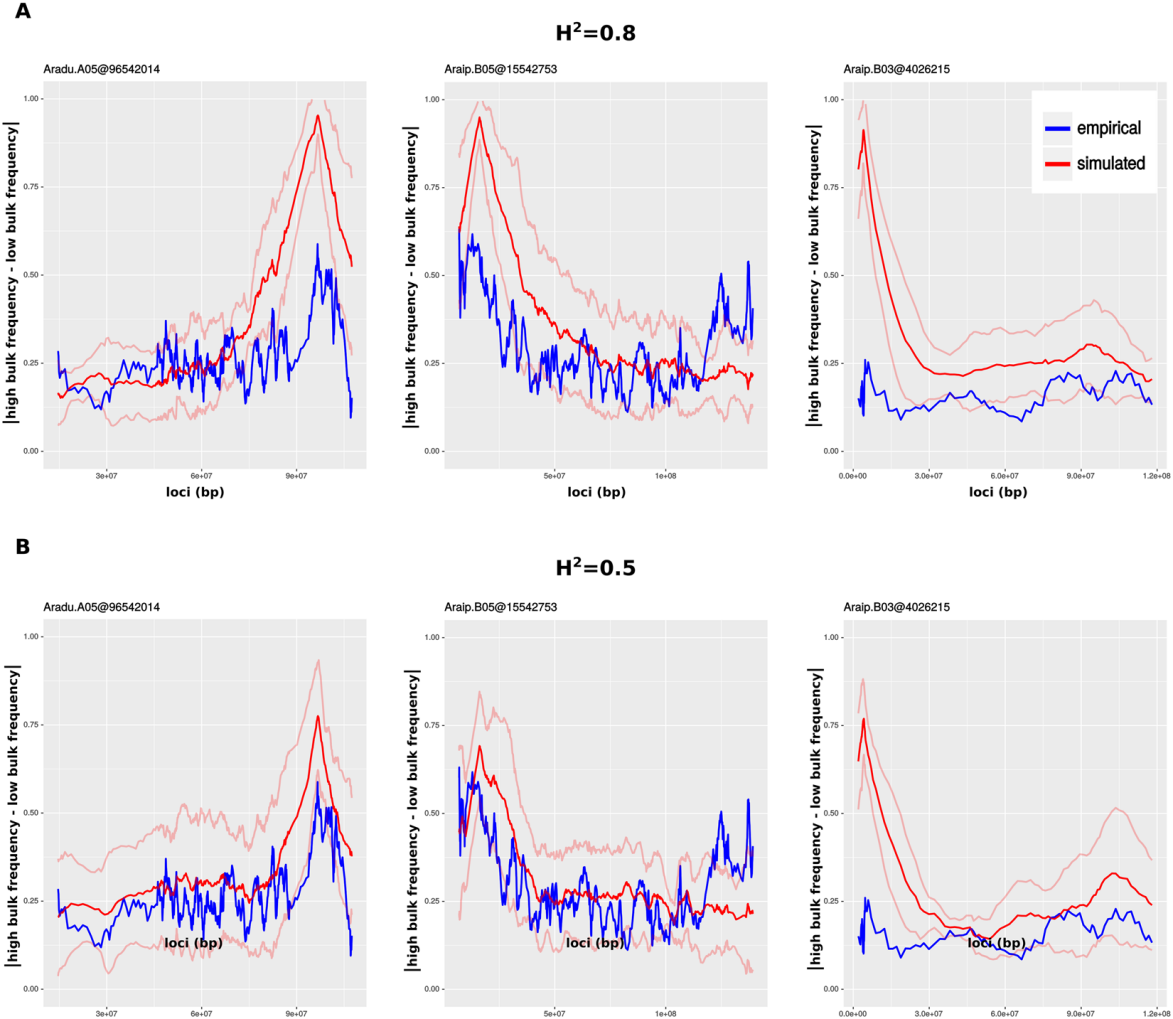
Empirical versus simulated QTL-seq results. The absolute frequency difference between bulks for QTL-containing chromosomes is plotted against genomic coordinates. The red, simulated data is based on 10 iterations in which parameters reflect those of the actual experiment. Blue lines reflect the empirical results. Bold lines represent the means and faded lines represent one standard deviation unit. (**A**) H^2^ = 0.8 (**B**) H^2^ = 0.5.

The empirical data revealed two strong peaks at chromosomes Aradu.A05 and Araip.B05, and one weak peak at chromosome Araip.B03 correlated with late leaf spot resistance of peanut. We simulated both H^2^ = 0.5 and 0.8. The study used peanut genotypes SPT06-06 as a resistant parent and Florida07 as a susceptible parent with 5,513 markers segregating. A population of 166 individuals was advanced to F6. The highest and lowest 5% was bulked for QTL-seq analysis. A simulation was run using the same parameters, which showed similar trends for the three peaks (Fig. 4A). However, lower-H^2^ results more closely resembled the empirical data (Fig. 4B). Heritability in this study was originally reported on a line-mean basis as 0.8, thus the better fit with simulation of H^2^ = 0.5 is expected given that we apply heritability at the level of individual replicate. In addition, we used equal effect sizes, while the empirical data indicate at least one QTL (on Araip.B03) is quite minor.

### “Levels” in *crossword*: genotypic replication in phenotype estimation and genomic selection

A major aim of *crossword* is to supply a flexible but powerful syntax for the exploration of realistic breeding strategies. Previous simulation frameworks often underestimate the importance of how particular genetic pools get advanced and evaluated and, therefore, underestimating the impact of factors such as residual heterozygosity and family size on genetic variance and phenotyping accuracy. For example, bulk advancement versus single-seed descent can have a substantial impact on the logistics of variety management. Some population level processes can be modeled using scripting libraries such as simuPop, SliM, etc.^12,13^, but scripting requires a general proficiency in programming and, more importantly, is not designed for dealing directly with controlled crosses. We have addressed this operational gap by incorporating the concept of “levels” into many *crossword* functions. Where applicable, users can specify one of four levels: “individual”, “family”, “cross”, or “population”. All populations have “individual” and “population” levels, but other levels depend on how the population is created and managed (See Fig. S2). For example, a researcher may want to create a population by crossing one individual by three other lines and then advancing the entire population in bulk. Alternatively, they may want to advance each cross as a separate bulk. The level parameter in *crossword* allows these variations with very minor changes when calling the “advance” function.

The “level” concept carries over into phenotyping and selection. Generally, the costliest aspect in genetic mapping and breeding is phenotyping. Unless clones or double haploids are used (which *crossword* supports), nearly all phenotypic replication of a “genotype” is actually based on a population of closely related individuals, usually referred to as a “recombinant inbred family”. *Crossword* gives users the power to fine-tune this family-creation function in order to precisely explore the implications of sample size on their final result.

**Figure 5:**
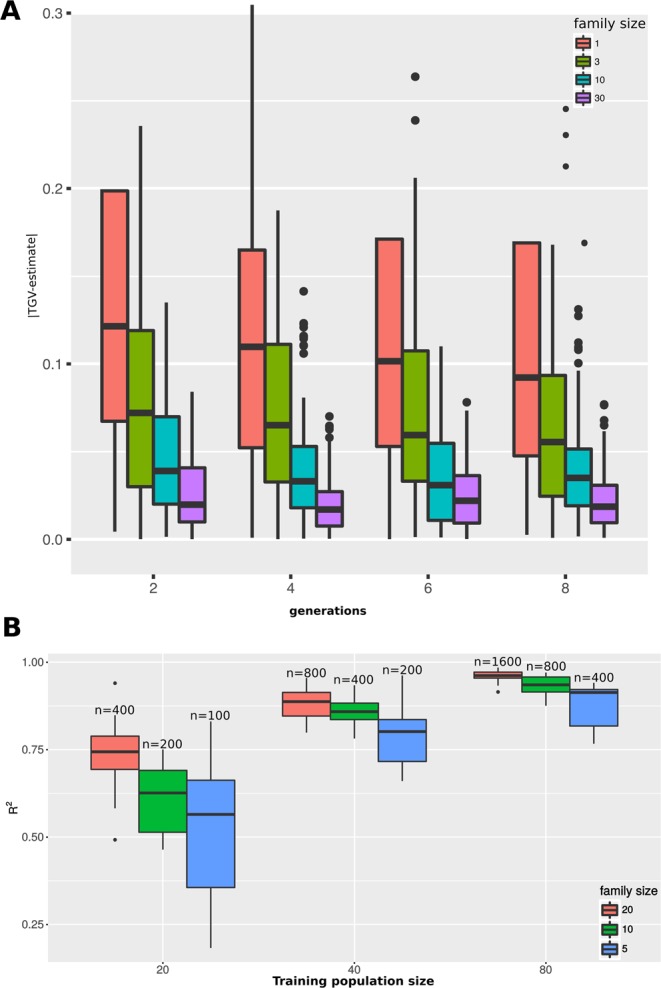
Impact of family size on experimental design. (**A**) Genomic Estimated Breeding Value (GEBV) as a function of family homogenization and replication. Boxplots depict the absolute difference between the TGV and the phenotypic estimate under a given set of parameters. The number of selfed generations are used to divide groups along the X-axis and 4 different family sizes are shown for a given generation. (**B**) Exploration of phenotyping accuracy versus training population size. Boxplots depict the prediction accuracy as an R^2^ value between the TGV of an individual and the GEBV of that individual. Models used to produce GEBV are based on the mean performance of families derived from individuals that are full-sibs of the test population.

To further illustrate the level concept, we explored the trade-off between time, field space/resources, and phenotypic accuracy. We simulated multiple combinations of generations and family sizes (Fig. 5A). For each line we compared the estimated true genetic (TGV) value calculated from family replicates to the TGV of the family progenitor. As expected, the accuracy of TGV improves as a result of both family homogenization - generations of selfing - and family size. Still, the degree to which these factors interact and the optimal balance between accuracy and expense naturally depends on the constraints of the research/breeding program. Clearly, there are diminishing returns of phenotypic accuracy as the number of replications goes up, but this diminishment is a function of generations.

For some traits, phenotypic replications involve the measurement of individual plants. Alternatively, some traits, such as yield, are replicated as rows containing numerous plants. Thus, a few generations of advancement with bulking would be required in order to achieve the replication numbers of 10 and 30. If needed, *crossword* can easily simulate this possibility by advancing at the family level as opposed to the individual level (see Fig. S2).

Genomic selection (GS) is another area in which phenotypic accuracy is crucial. GS was developed within the cattle breeding community as a way of predicting complex traits solely from marker-based estimates of kinship to a phenotyped training set^14^. Many crop breeding programs are now exploring best practices for the implementation of GS^15^. In this context, one under-explored benefit of working in plants is the ability to phenotype genotypes over multiple years and locations to dramatically improve the estimate of the True Genetic Value (TGV). As with family homogenization and family size, optimal genomic selection logistics are additionally complicated by the need to determine the training population size that will result in the highest prediction accuracy given the available resources.

We simulated three different training population sizes and three different family sizes within each set (Fig. 5B). “QTN_random” phenotyping method was used to sample 60 QTNs for each one of 10 iterations, and the QTN effect was assigned using the gamma distribution. Populations were advanced to F5. We evaluated prediction accuracy as the R^2^ between the TGV of an individual that has full-sibling relatedness to the training set and its phenotypic prediction based on the trained model, “kinship.BLUP” function of rrBLUP was used (Table S1)^16^.

Larger training set size is correlated with improved prediction accuracy, although, at 400 replicates, sizes of 40 and 80 are roughly equivalent (Fig. 5B). Thus, it may be more appropriate for some species to have smaller training sets and larger family sizes depending on the species’ reproductive limitations. Even in large training sets, family size has a substantial impact, raising the median R^2^ by 10% with 5 extra replicates while also reducing the expected variation. Still, based on these results, as a “rule-of-thumb”, breeders should favor large training sets over within-family replicates.

### QTN polarity has a substantial impact on effect estimation of multigenic traits

In terms of simulating phenotypes, important varieties generally have some extreme trait that sets them apart. For example, one variety may be highly resistant to disease relative to another highly susceptible variety^11^. Pre-existing platforms rarely support the ability to easily simulate this reality. In *crossword*, QTN selection, aside from being supplied or random, can be biased towards one parent containing predominately positive or negative alleles. For mapping a small number of large effect QTN, this bias may be fairly irrelevant, but, as the number of QTN increases, this scenario could substantially impact QTL detection and effect estimation.

We simulated four scenarios that highlight the consequences of asymmetrical effect polarity. In one set, 3 QTN and 15 QTN had randomly polarized effects (“QTN_random”) that are roughly evenly distributed between parents. Alternatively, in the second set (“highLow”), all effects are positive in one parent and negative in the other. For these simulations, we used a H^2^ of 0.7 and a population of 200 individuals advanced to F6 generations before phenotyping 200 families of 5 individuals (in effect, replicates) apiece.

**Figure 6:**
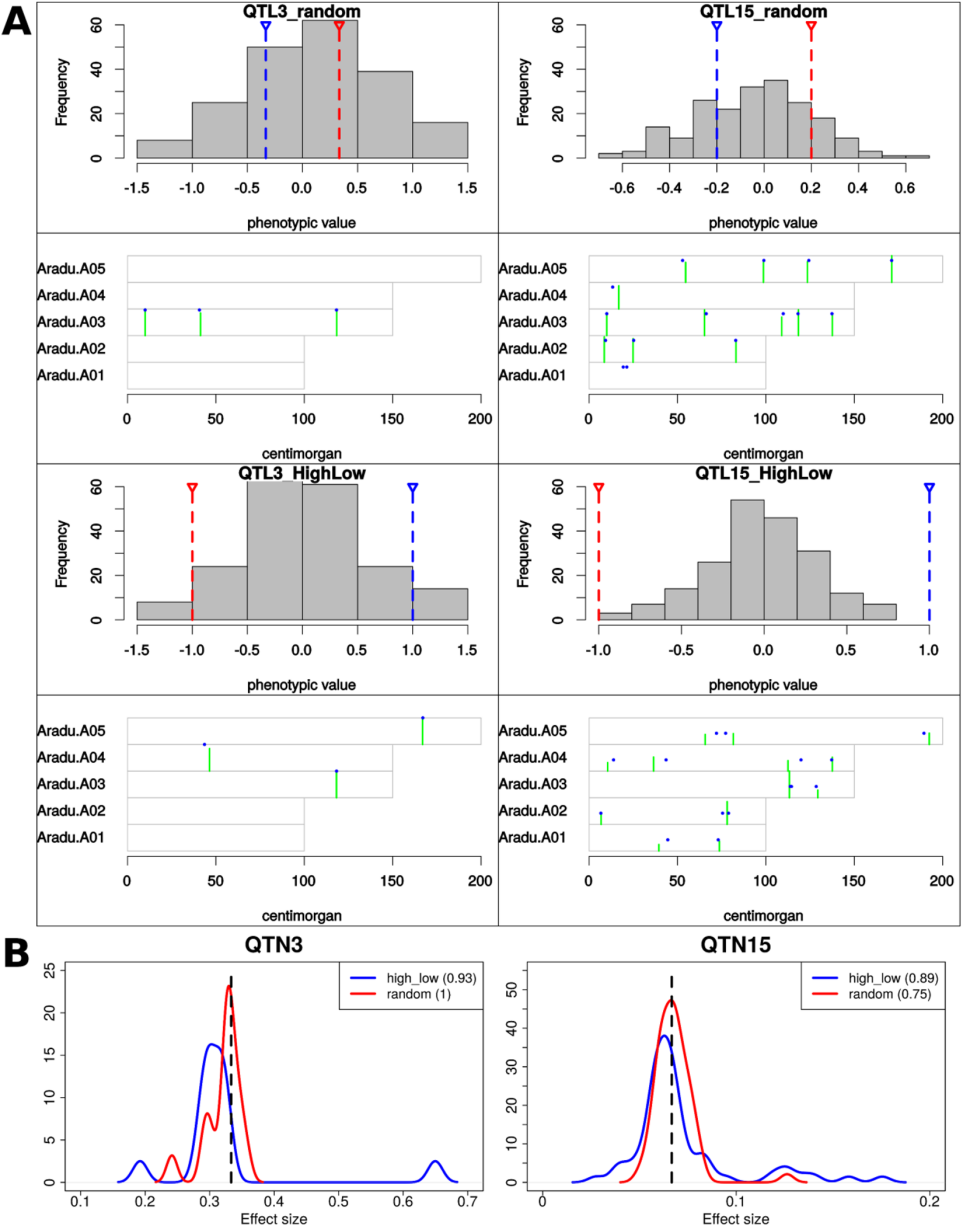
(**A**) Impact of random versus parentally-biased allelic effects. Each panel shows the phenotypic distribution of the mapping population and a chromosome plot of the simulated QTN position relative to the mapped position. In the distributions, high (red) and low (blue) parent values are indicated. In chromosome plots, blue dots indicate the simulated positions and effect sizes. (All the effects are equal.) Green bars indicate the estimated QTL position and the effect size. Chromosome maps are scaled in the y dimension to the largest effect size across all estimated effects across all chromosomes. (**B**) Distributions of effect sizes across all iterations for highLow phenotyping methods with 3 and 15 QTN. The dotted vertical lines represent the actual effect for all QTN in the simulation, H^2^ was adjusted to reflect the heritability of entry mean basis. (3 QTN simulations have higher effect sizes than 15 QTN because maximum TGV in both cases is equal to 1).

As expected, in both cases of 3 QTN, the polarity of the effects was generally irrelevant due to the fact that QTN are rarely in strong linkage with one another (Fig. 6). (Here we differentiate “QTN”, i.e. simulated causal variant, from “QTL”, i.e. estimated genomic region and effect.) As linkage becomes a factor with 15 QTN (~3 QTN per chromosome), effect estimates tend to vary more dramatically (Fig. 6B). In the case of random polarity, fewer QTL should be detected because effects in the same linkage block will cancel each other out, creating the appearance of weaker, harder-to-detect QTL (Fig. 6A, top-right, “Aradu.A01”). This expectation is borne out in simulation, where the smallest fraction of QTN (75%) were discovered in the 15 QTN “random” mode. Alternatively, when QTN have biased polarity in one parent (“highLow”), the QTN are more frequently detected (89%), and those missed are generally combined into a phantom peak (Fig. 6A, bottom-right, “Aradu.A02” and “Aradu.A03”). This combining is reflected in the fact that estimates are generally higher and some substantially exceed the actual effect size. Even in “random” mode, with 15 QTL, effect estimates can exceed the actual but this is a rare occurrence and never to the degree observed using biased parents. As shown, precise expectations for mapping an oligogenic trait can benefit from the incorporation of parental bias, particularly as the number of QTN per chromosome exceeds 3.

## Conclusions

We introduce a breeding simulation platform, designated *crossword*, which advances the ability to simulate genetic and breeding experiments in the following ways:

- The structure of founder populations is rarely as uniform as those produced by coalescent simulation. *crossword* uses actual structures derived from genetic variation data and supplied as VCF or Hapmap files (Fig. 2).
- Recombination is never uniform across the genome but this discontinuity is difficult to apply in simulation if a genetic map is unavailable. Even when maps are available, conversion to genomic coordinates is cumbersome. *Crossword* offers a shortcut to users by exploiting the general correlation between gene density and recombination as well as the availability of a GFF gene-annotation file for most organisms undergoing sustained breeding efforts (Fig. 3).
- Genetic experiments and breeding typically involve highly structured populations based on crosses and subfamily relationships. This structure is built into *crossword* in order to dramatically simplify the description of complex simulations through the use of levels (Fig. 5 and associated text).
- Parents with highly divergent phenotypes are often used to genetically map traits. *Crossword* offers the ability of easily apply such bias when simulating the genetic architecture of a trait (Fig. 6). In addition, *crossword* realistically models heritability when multiple rounds of selection occur in a single simulating (see Methods).
- Generally, geneticists and breeders are not computer programmers. *Crossword* is designed to cater to numerous programming proficiency levels and offers a graphical interface as an entry point into the platform (Fig. 1). That said, by using available genomics data, *crossword* still allows for very sophisticated and realistic applications (Figs 1 and 2).

## Methods

### Genetic Architecture

It is assumed that causal variants are generally located in or near genes. Instead of randomly picking positions across the genome, *crossword* can use the gene density information of the input annotation file (gff) to bias randomization toward the regions having more genes.

The genetic architecture of traits is simulated in one of three modes: *QTN_random*, *QTN_supplied*, or *high_low_parent*. In *QTN_random* mode, each QTN (Quantitative Trait Nucleotide) position is drawn at random across the genome and underlying allele effects are assigned based on the supplied distribution. In *QTN_supplied* mode, positions and the effects associated with alleles are defined by the user. In *high_low_parent* mode, positions are drawn at random, as in *QTN_random* above, but allelic effects are biased to be positive in the user-specified “high” parent and negative in the “low” parent. True genetic values (TGVs) are then summed for a particular genotype. (If QTN are dominant, the TGV and true breeding value will be different.) Epistatic interactions are currently unsupported.

In its simplest form, heritability (H^2^) is the relationship between genetic variance (V_G_) and residual variance (V_R_). V_G_ can change substantially depending on the parents in a cross and downstream selection in a population, among other things. Alternatively, V_R_ will be essentially constant assuming individuals are evaluated in a consistent set of environments. The common approach in many simulation frameworks of calculating V_R_ from the supplied H^2^ for every phenotyping step is therefore conceptually dubious, since V_G_ and V_R_ are not expected to show substantial covariance. Indeed, in simulation of long-term selection experiments, such a conventional approach will dramatically overestimate the ability to accurately select superior genotypes in late generations. To that end, in *crossword*, the user supplies either “absolute” or “average” H^2^. “Absolute” H^2^ is the heritability in a theoretical cross that segregates for all possible QTN. “Average” H^2^ considers the average number of QTN segregating in all possible crosses between founders. (If there are only two founders, then the modes are equivalent because only segregating SNPs will be selected as QTNs.) In these theoretical crosses, we assume a very large population size, free recombination among loci, and complete homozygosity. Dominant effects are irrelevant in this calculation. V_R_ is then calculated using a set of 10,000 individuals randomly selected from all possible combinations of individuals binomially distributed for positive and negative alleles. The subsample approach is used to simplify calculations when effects are not equal and/or do not follow a formal distribution, such as in used-supplied mode. The actual H^2^, including dominance effects, for a population is reported whenever that population is phenotyped, such that the user can modify the supplied H^2^ if inappropriate.

### Founder haplotypes and recombination

In heterozygous founders, we assume that the haplotype phase is unknown and randomly select one allele at heterozygous SNPs to belong to one homolog. Heterozygous sites in founders are discarded when homo = TRUE, which is default behavior because heterozygosity in highly inbred founders is often a genotyping error. When homo = FALSE, heterozygous loci are randomly phased into parental haplotypes. Recombination between founder haplotypes is simulated using the external R library, simcross [http://kbroman.org/simcross/] (Table S1), which takes into account interference (m = 10, by default). By default, at least 1 chiasma per meiosis is required. Fig. S1 shows the genomic loci of crossing-over across different population sizes.

Founder haplotypes are imported in either HapMap or VCF formats. The names of founders in these files can be used directly in *crossword* to define parents. As with most contemporary simulations^7^, only haplotype structure is simulated under a given set of crossing/advancement steps. When phenotyping or genotyping is performed, physical-to-genetic distance functions are used to produce genotypic information for each haplotype. The relationship between genetic and physical distances is known to be nonlinear for the vast majority of species^8,9^. In *crossword*, we use the known relationship between gene density and crossover frequency to produce a function that can be used as a substitute for explicit genetic-distance data (Fig. 3). GFF files are supplied to *crossword*. A sliding window is used to calculate gene density versus physical distance. The R “loess” function is fit to the resultant relationship, and this function is used to convert physical coordinates into genetic coordinates based on total genetic length (cM) supplied by user per chromosome (or 100 cM for all chromosomes if values are not supplied).

Halotypes are converted to genotypes, using the transform function above, when a genotyping or phenotyping set is required. Given the computational demand on this step, *crossword* uses the Rcpp external R library and custom C++ programs to optimize the speed of operations ~60-fold as a part of “get_parental_genotypes” function. The script recognizes and reads the VCF or hapmap files, differentiates homozygous/heterozygous/indel loci, reformats the output and then applies it to a prediction function to estimate the genomic coordinates from genetic coordinates.

### Genetic variance in founder population versus single cross

All polymorphisms in the input founder file (VCF or hapmap) are used to select QTN. Therefore, when making a cross, the actual number of segregating QTN will be lower if the founder file contains more than the two individuals in the cross. This approach allows users to apply genome-wide association data from the diversity panels that they may be using as founders and to accurately simulate the reduction in relative genetic variance that occurs in derived populations. A helper function is supplied to assist in reducing VCF files depending on the level of polymorphism desired.

### QTL identification and effect estimates for biparental crosses

In simulations (Fig. 6), QTL were mapped using r/qtl^17^. To focus on effect estimates, the true genetic positions for all markers were used directly as opposed to being estimated from recombinants. Using the ‘stepwiseqtl’ function, the maximum number of QTL to discover (“max.qtl”) was iteratively increased until the minimum LOD score for every discovered QTL was <3. Effects were assessed based on the final resultant model. The R script to perform mapping is available in the auxillary_scripts folder released with *crossword*, but depends on r/qtl, which is not included with *crossword*.

### QTL-seq simulation

Data and parameters for Fig. 4 are derived from^11^. The dataset contained 5,513 SNPs between the two peanut varieties SPT06-06 and Flordia07. QTL-seq analysis revealed three QTLs. *Crossword* offers the capability of simulating reads from a genomic sequence generated from a set of individuals in a simulation. Reads can then be simulated from these sequences if a user wants to explore a full genotyping-by-sequencing pipeline. Alternatively, since this read simulation approach can be computationally demanding, *crossword* offers an auxiliary program to add sampling and read coverage variation to a set of underlying genotypes. In actual experiments involving combining DNA from multiple genotypes as a single sample, such as QTL-seq, individual genotypes are obviously not known. Conversely, in simulations users can exploit this knowledge to get a realistic sampling of each bulked sample. In our simulation, we used an average coverage from the original experiment (30x across the bulk) and a standard deviation of 7. For each locus, a random variant was sampled from a normal distribution based on these values, and this random number of alleles was sampled with replacement from the set of all bulked genotypes.

### Source data

The genomic data of peanut and soybean was collected from PeanutBase [https://peanutbase.org] and Phytozome [https://phytozome.jgi.doe.gov], respectively. Further location details are described in *crossword* manual.

## Supplementary information

supplemental materials

## Data Availability

*Crossword* is available at https://github.com/USDA-ARS-GBRU/crossword. A detailed manual, function description file, and demo data are also available. Each simulation described in this manuscript is also available as an example (see https://github.com/USDA-ARS-GBRU/crossword/examples) identified by the figure to which it applies.
