# Supplementary material for: Crossword: A data-driven simulation language for the design of genetic-mapping experiments and breeding strategies
Source: Sci Rep. 2019 Mar 13;9:4386. doi: 10.1038/s41598-018-38348-y (PMC6416259; doi:10.1038/s41598-018-38348-y)
Supplement: Supplementary file 1 — supplemental materials [file 41598_2018_38348_MOESM1_ESM.pdf]

Crossword: A data-driven simulation language for the design of genetic-mapping experiments and breeding strategies

Walid Korani[1] and Justin N. Vaughn[2*]

[1]Center of Applied Genetics Technology, The University of Georgia, Athens, GA, 30602.

[2]United States Department of Agriculture, Athens, GA, 30602

* Corresponding author

phone: (865) 804-5182

E-mail: justin.vaughn@ars.usda.gov

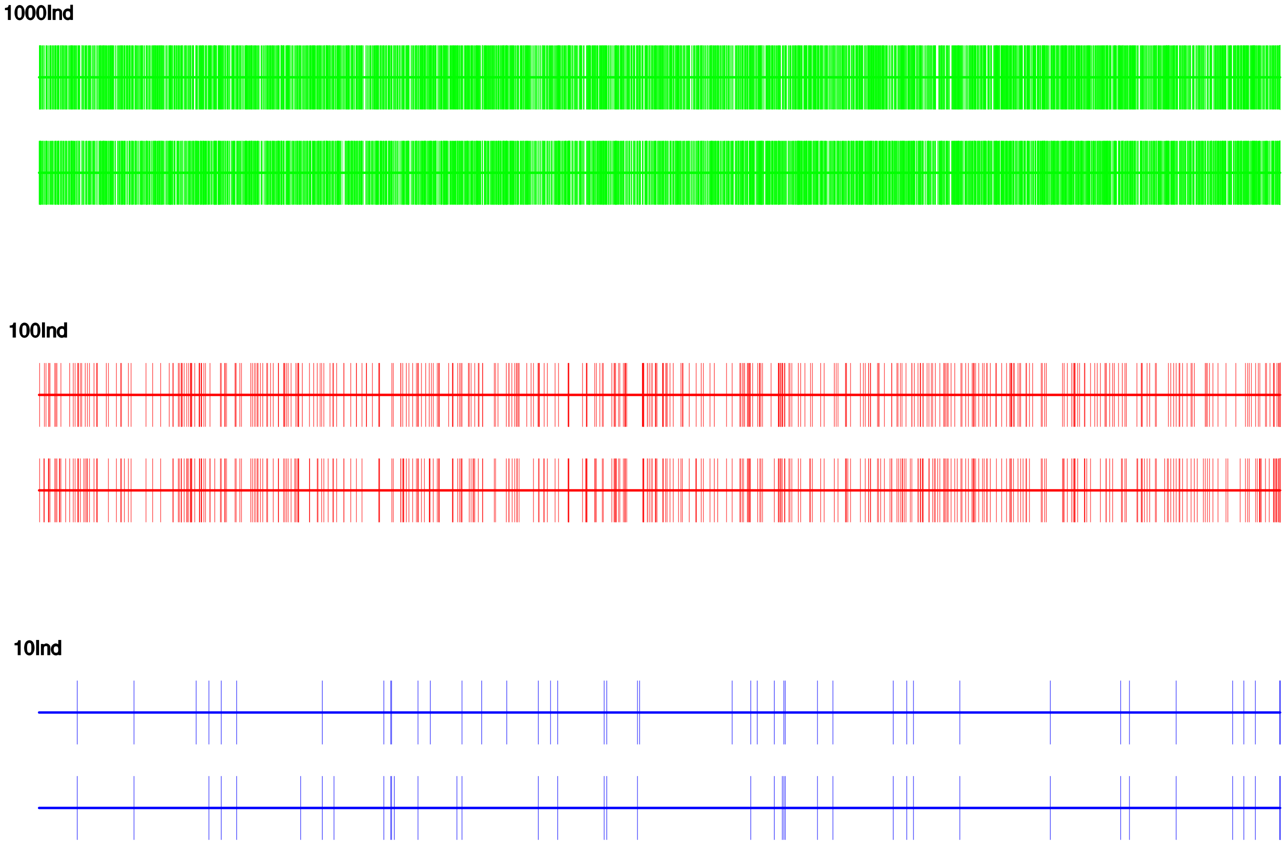

S1 Fig. ***Crossing*****-over loci for advanced generation of different population sizes.**

The concept of levels in crossword

Level = individual or family or cross or population

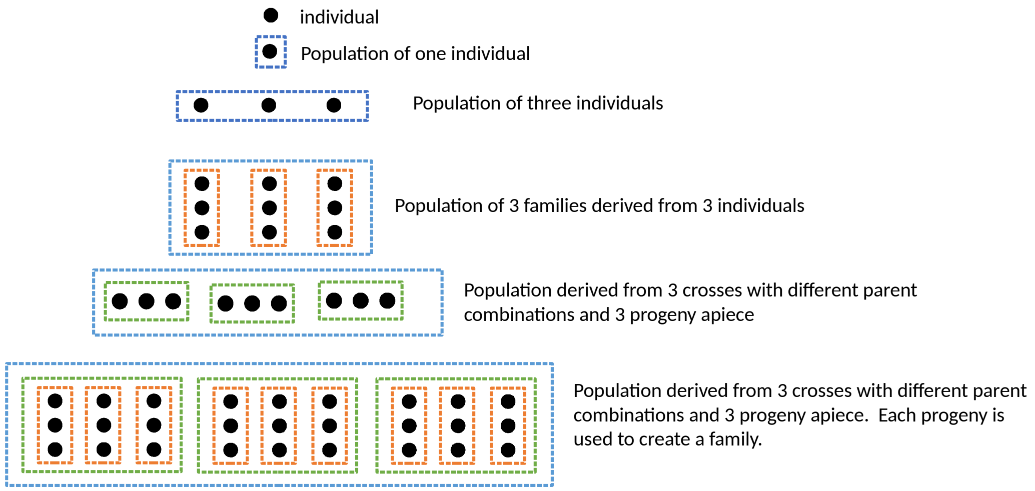

Standard biparental cross and recombinant inbred line (RIL) generation

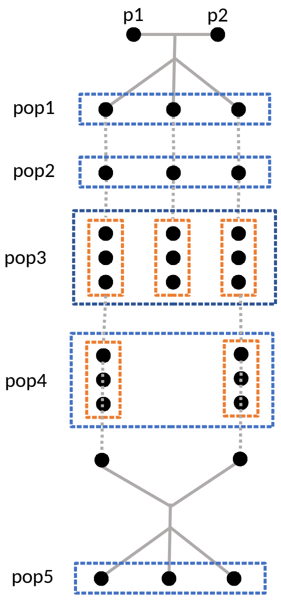


```
pop1 = cross(P1=p1, P2=p2, N = 3)
#simple biparental cross

pop2 = advance(pop = pop1, F = 6, level = "individual")
#advancement is by selfing with single-seed descent (level = "individual") for 6 generations
pop3 = createFamilies(pop2, S = 3)
#population is advanced an additional generation and related families are created

pop4 = select(pop = pop3, mode = “top”, N = 2, level = “family”)
#selection occurs at the family level by averaging across individuals
#only top 2 at this level are selected

pop5 = cross(pop4:1:1, pop4:2:1, N=3)
#new biparental cross
#After a selection function, pop4 is ordered by top family and then top
individual within a family.  This is a cross between the top individual
from each of the top 2 families
```

The effect of level value in selection and other functions

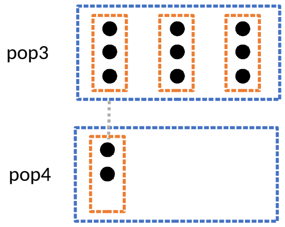


```
pop4 = select(pop = pop3, mode=”top”,  level = "individual" , N = 2)
#Selection occurs at the individual level because level = "individual"
#Only top 2 at this level are selected
#Family structure is still maintained - although there is only 1 family left in this example - unless
reduced as described below
```

Advancment without single seed descent (ssd) (e. g. "in bulk")

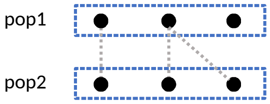

pop2 = advance(pop = pop1, F = 6, level = "population")
#advancement is by selfing for 6 generations , but not all lines are guaranteed to advance because advancement occurs at the population level (1) not the individual level (0) as with ssd
#random genetic drift can clearly occur depending on population size

Advancment in bulk with obligate outcrossing

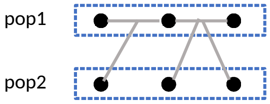

pop2 = advance(pop = pop1, F = 6, level = "population", clevel = "population")
#advancement is by obligate outcrossing for 6 generations
#The outcross frequency is set to 1 as a global parameter in the run file, so it does not get set in individual functions
#With populations that have higher levels of structure, outcrossing can be limited to a particular level as you might see with barriers between families planted in a field. Note that outcrossing can occur at the cLevel given as well as lower levels, so ocLevel = "population" would allow outcrossing at "family" level as well.

Advancment by ssd with partial outcrossing

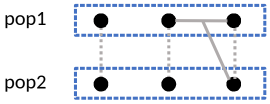

pop2 = advance(pop = pop1, F = 6, level = "individual", clevel = "population",)
#advancement is by ssd but some outcrossing can occur. As in breeding, id will follow the mother plant, indicated by dotted line
#As above, the outcross is set as a global parameter in the run file, so this function arguments are identical to above, it is only the global outcross frequency parameter that would be different in the run file.
#Some heterozygosity is maintained

Nested population creation and manipulation

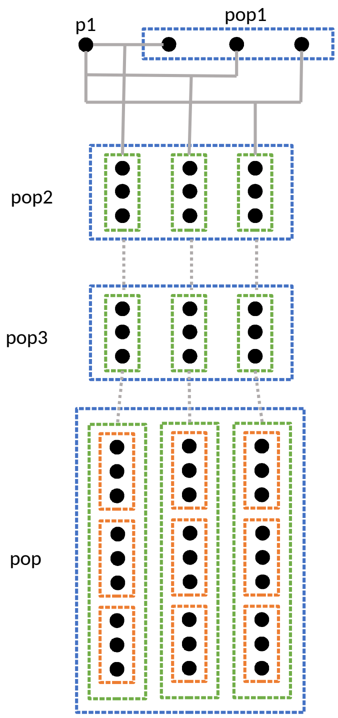

pop1 = cross(p1, pop1, size = 3)
#each cross results in a population of 3 individuals

pop3 = advance(pop = pop2, F = 6, clevel = "individual" , level = "individual")
#advancement is by selfing with single-seed descent for 6 generations

pop4 = createFamilies(pop = pop3, S = 3)
#population is advanced an additional generation and related families are created

S2 Fig. *Crossword* syntax overview with graphical examples.

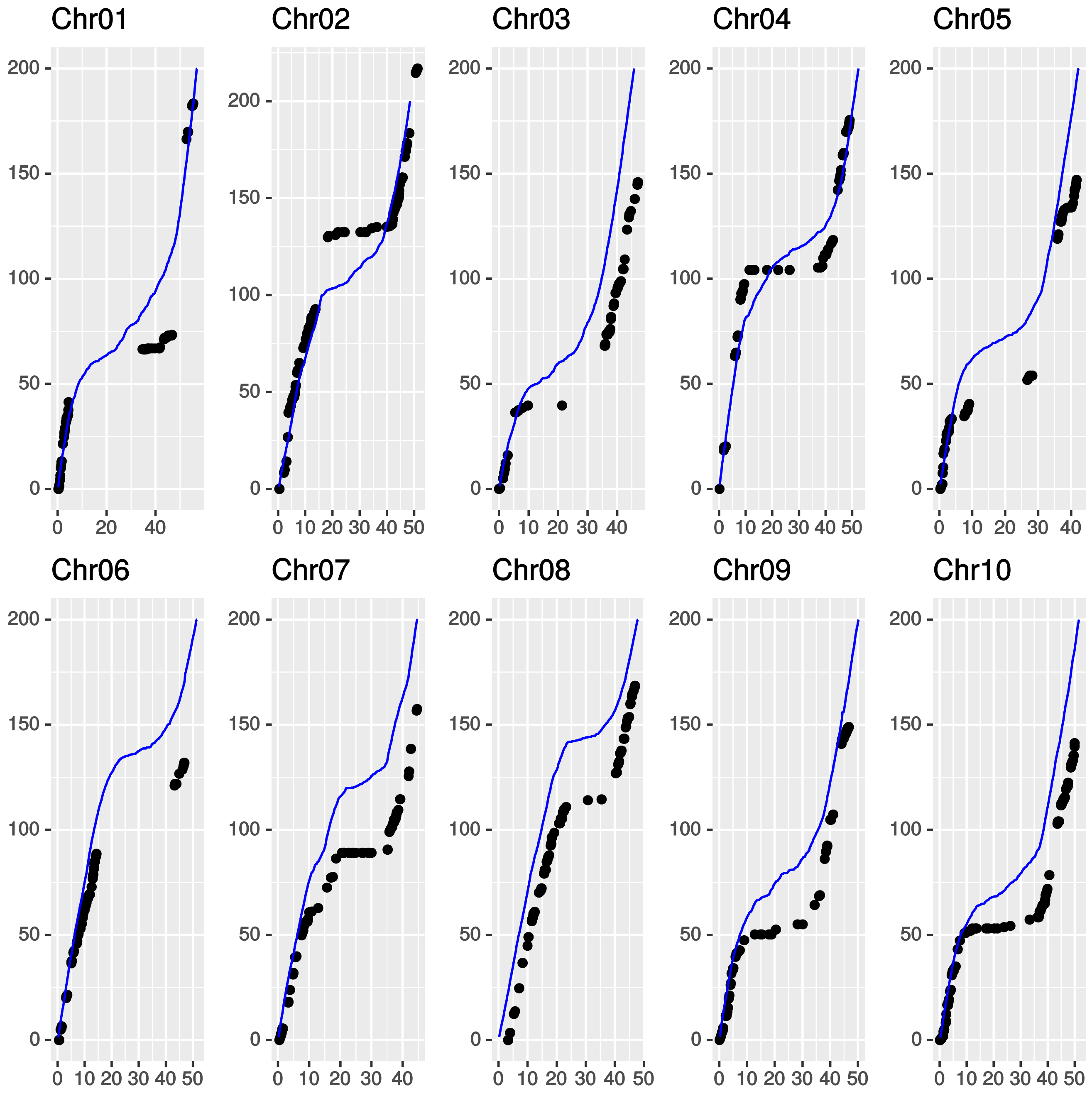
Chr01
Chr02
Chr03
Chr04
Chr05
Chr06
Chr07
Chr08
Chr09
Chr10
0
50
100
150
200
0
20
40
10
30
50

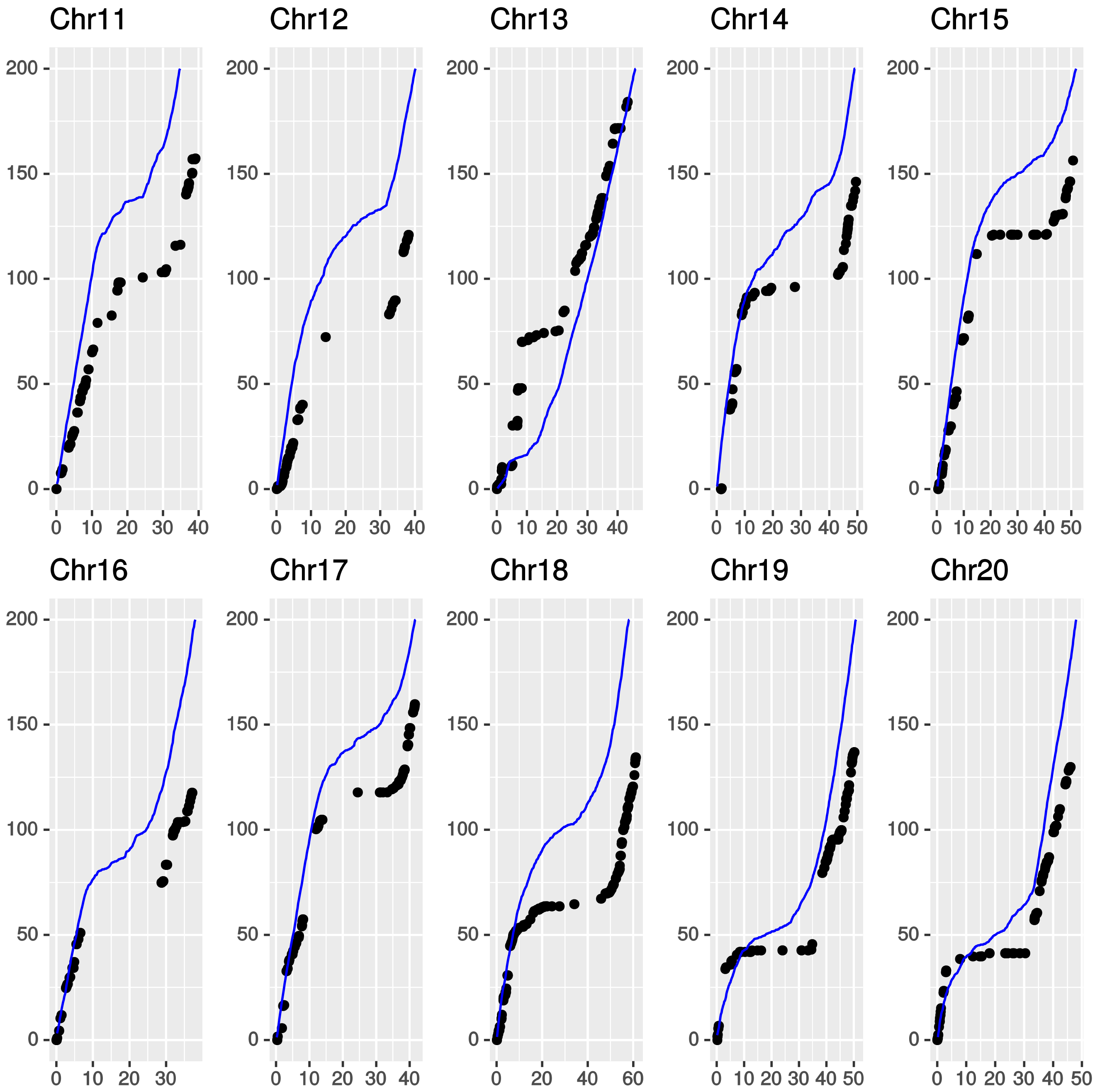

Chr11
Chr12
Chr13
Chr14
Chr15
Chr16
Chr17
Chr18
Chr19
Chr20
0
50
100
150
200
0
10
20
30
40
50
60

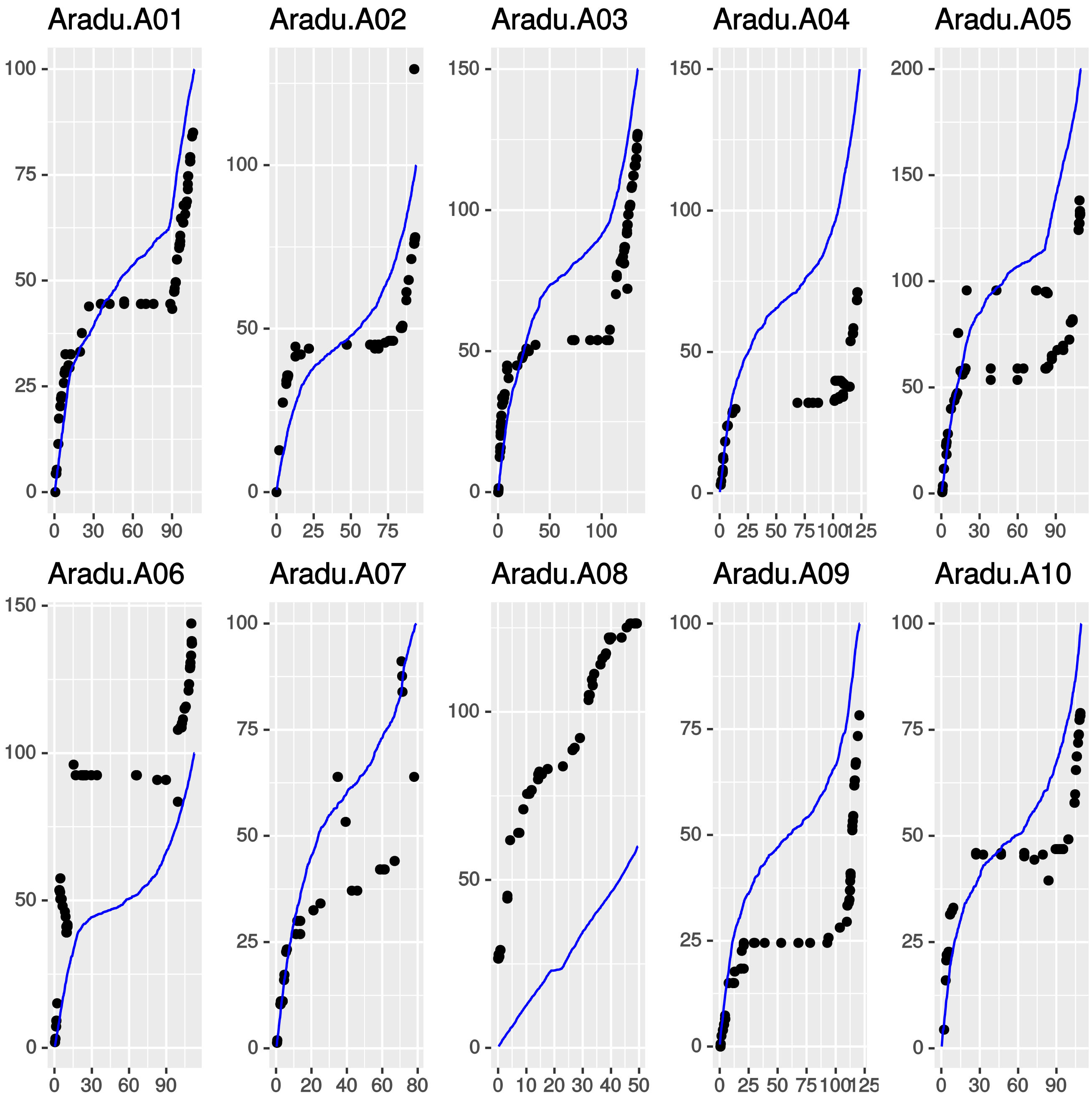

Aradu.A01
Aradu.A02
Aradu.A03
Aradu.A04
Aradu.A05
Aradu.A06
Aradu.A07
Aradu.A08
Aradu.A09
Aradu.A10

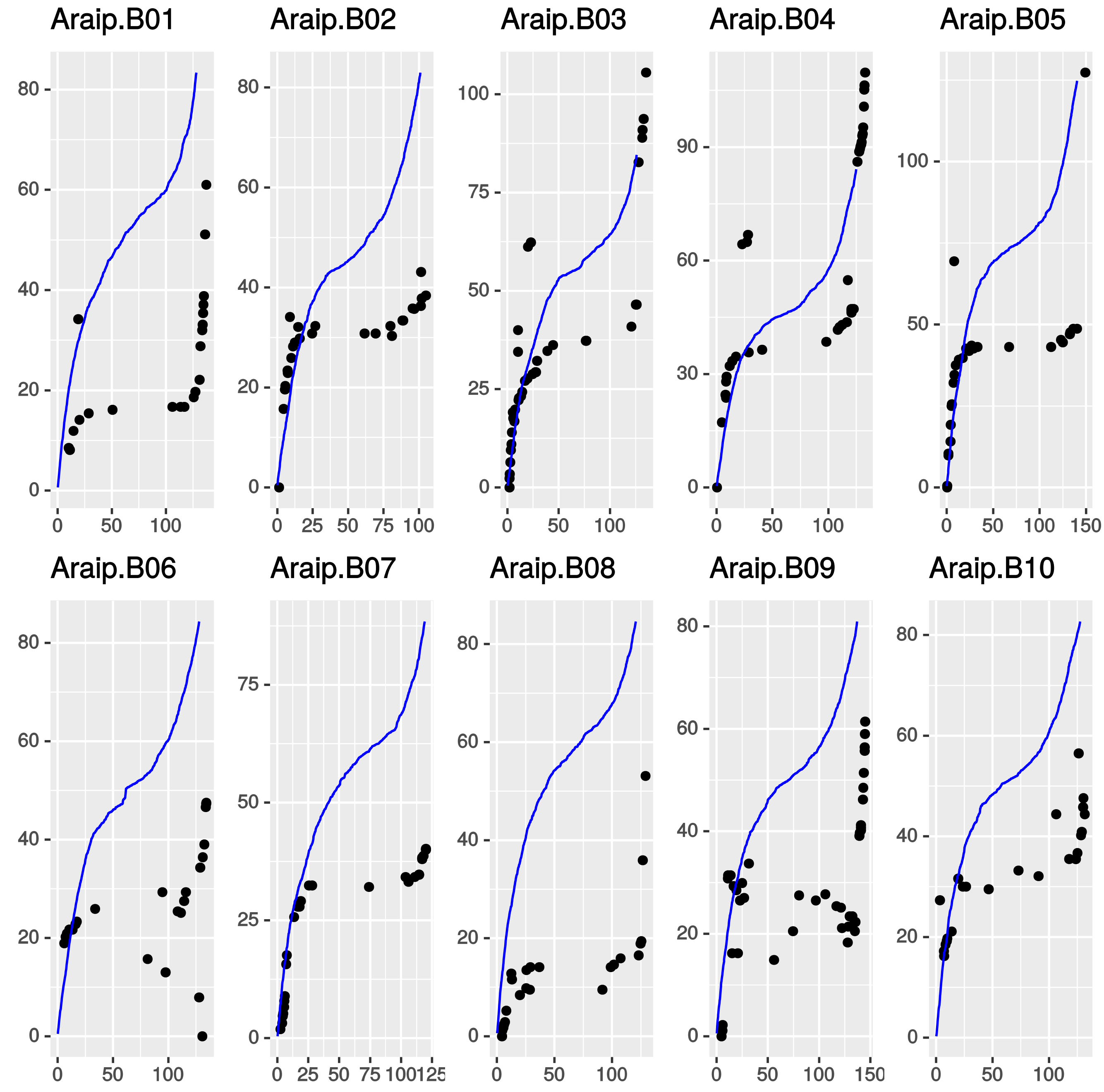

S3 Fig. Plots of recombination modeling in *crossword*

S1 Table. External resources

External Resource	Reference	Licence
simcross	http://kbroman.org/simcross/<br>https://kbroman.org/simcross/assets/vignettes/simcross.html	Open_Source
rrBLUP	Endelman, J. B. 2011. Ridge Regression and Other Kernels for Genomic Selection with R Package rrBLUP. Plant Genome 4:250-255	Open_Source
ART	Weichun Huang, Leping Li, Jason R Myers, and Gabor T Marth. ART: a next-generation sequencing read simulator, Bioinformatics (2012) 28 (4): 593-594	Open_Source
